# Thoughts on and Motives for Leaving the First-Line Manager Position: A Qualitative Study

**DOI:** 10.1155/jonm/9906205

**Published:** 2025-08-16

**Authors:** Jonas Svanström, Magnus Lindberg, Maria Lindberg, Bernice Skytt

**Affiliations:** ^1^Department of Caring Sciences, University of Gävle, Gävle, Sweden; ^2^Centre for Research and Development, Region Gävleborg, Uppsala University, Gävle, Sweden; ^3^Department of Public Health and Caring Sciences, Uppsala University, Uppsala, Sweden

**Keywords:** authority, first-line managers, qualitative research, resign

## Abstract

**Aim:** The aim of this study was to describe first-line managers' thoughts on and motives for leaving their position.

**Introduction:** The turnover rate of first-line managers impacts healthcare organizations' overall performance. Burnout, dissatisfaction, and desired career advancement contribute to the intention to leave. High turnover negatively impacts patient care and has financial consequences. Involving managers in decision-making, providing support, and addressing workload and resource challenges are known to be crucial factors in retaining managers.

**Method:** Sixteen semistructured interviews were conducted with first-line managers. Qualitative content analysis was used to analyze the data.

**Findings:** Our study showed that some first-line managers experience difficulties at work, leading them to reach a breaking point and leave. These difficulties stem from a lack of influence, feeling unheard, and having inadequate support. Some first-line managers reach a point where they feel the need to move on, often due to personal reasons or a lack of motivation. Upon deciding to leave, managers commonly have a plan to ensure a smooth transition for their successor, aiming to help both the new manager and the unit's staff.

**Conclusion:** First-line managers can perceive their work situation as challenging and frustrating when they lack authority and support. This can lead to them leaving their role for reasons such as retirement, seeking more time for family, or seeking new work challenges. It is important for healthcare management to empower and support first-line managers. Smooth transitions for successors and staff are crucial, regardless of why a manager leaves.

## 1. Introduction

The importance of the first-line manager's role in health care is well established through research. Health care has been facing several challenges, and one recurring issue is the turnover rate of first-line managers. A systematic review by Brown et al. [1] identified several key factors affecting retention of first-line nurse managers. These include organizational elements such as commitment, trust, resources, administrative systems, and role-related aspects like support, empowerment, manageable expectations, and work–life balance. Personal considerations, including feeling valued, job alignment, and family commitments, also play a significant part. Furthermore, Keith et al. [2] found that job satisfaction among first-line managers, which is influenced by workload, organizational support, and relationships with supervisors, affected retention in health care.

The turnover rate of first-line managers is a common problem in many healthcare organizations. A recent study indicated that over half of all first-line managers had the intention to leave their current position within the next 5 years [3]. Previous studies have shown that the most commonly reported reasons underlying first-line managers' intentions to leave their position are burnout [3, 4], desired career advancement within or outside the organization, and retirement [3, 4]. Dissatisfaction with the job and organizational factors, such as reorganization, lack of support, low salary, insufficient authority, or poor relationships with superiors, might also contribute to first-line managers' intention to leave their role [5].

First-line managers serve a vital function in fostering a positive work environment that supports staff retention and satisfaction [6]. When first-line managers leave their position, this can have significant consequences for both staff and patients. Cummings et al. [7] emphasized that first-line managers through their leadership can foster higher job satisfaction and better relationships between staff members. In a study by Warshawsky et al. [8], high turnover among first-line managers had a negative impact on patient health, as patients were more likely to develop pressure ulcers compared with patients at units with no turnover, owing to loss of continuity and competence among staff. Furthermore, research by Warshawsky et al. [9] highlights that first-line managers' experience in boosting competence and improving nurses' work environment leads to lower rates of missed care and higher quality of care. In addition to clinical outcomes, the resignation of a first-line manager has economic implications as the cost of replacing can reach up to 75% of the manager's annual salary [10].

In their role, first-line managers oversee staff, manage administrative tasks, and facilitate communication in the ever-changing healthcare landscape [11]. First-line managers also face the challenge of having full economic responsibility for their unit's budget, despite often feeling excluded from the budgeting process [12]. First-line managers are responsible for ensuring the efficient operation of their unit, but their decision-making authority is usually limited. Involving first-line managers in decision-making and providing them with information is essential for them to fulfill their responsibilities [11]. When first-line managers are excluded from decision-making, this can erode trust and prevent them from performing their work effectively [5]. Moreover, the support and trust of superiors can play an important role in a manager's decision to stay in or leave their position [3, 13].

In recent years, health care has faced numerous challenges, including staff shortages, budget constraints, and a global pandemic. As a result, first-line managers have encountered difficulties in managing their resources effectively while still providing high-quality healthcare services to patients. In some cases, first-line managers prioritize their staff's needs over their own. In one study, several participants underlined the importance of personal interactions and providing support to staff, even if this meant working overtime or neglecting their own administrative tasks [14]. A study conducted by Cregård and Corin [13] identified both push (leave) and pull (stay) factors within the organizational structure, as well as job characteristics that could affect public sector managers' turnover. For instance, having a large number of subordinates was a push factor, whereas having fewer subordinates was regarded as a pull factor in the organizational structure. In terms of job characteristics, insufficient control over one's own agenda was a push factor, whereas having a sense of control was an example of a pull factor [13].

For several reasons, it is important to understand the motives of first-line managers who leave their managerial role. First, first-line managers are considered to be the backbone of any organization, responsible for managing a team of employees and ensuring that daily operations run smoothly. Therefore, their leaving can have a significant impact on overall performance. Second, identifying the reasons why first-line managers leave can help organizations address any issues or gaps in their management practices that may contribute to high turnover rates. Lastly, gaining insights into the motivations of first-line managers who leave can help organizations develop effective strategies to attract and retain first-line managers. Prior studies have primarily focused on first-line managers' intentions to leave their position. However, there is a lack of comprehensive understanding regarding the specific factors that prompt managers to act on such an intention. Furthermore, existing qualitative research on first-line managers who have left their role is dated, creating a need for up-to-date insights in this area. This study seeks to address these gaps by investigating the specific thoughts and motives that lead first-line managers to leave, thus hopefully contributing to a deeper understanding of turnover in first-line managerial positions. Such research is important for organizations, as high turnover among first-line managers results in decreased productivity, increased costs for recruitment and training, and negative patient health outcomes. Organizations may be able to develop targeted retention strategies by understanding the specific thoughts and motives behind resignations, ultimately fostering a more stable and effective workforce. The aim of this study was to describe first-line managers' thoughts on and motives for leaving their position.

## 2. Method

### 2.1. Design

The approach of this study was qualitative with a descriptive design. The manuscript was prepared using the Standards for Reporting Qualitative Research (SRQR) reporting guidelines as a checklist [15].

### 2.2. Setting and Sample

This research was conducted in a Swedish region with a population of just below 300,000. The region encompasses both urban and rural areas, with hospitals and primary care services accessible throughout. The healthcare system in this region is structured into multiple operational areas. Each operational area is supervised by a second-line manager responsible for several first-line managers and, in some cases, one or more assistant managers. A human resource strategist and an economist collaborate closely with the management team in each operational area. In the Swedish healthcare system, managers are usually hired on fixed-term contracts that can be extended after consultation with the employer. Sweden's statutory retirement age ranges from 62 to 68 years, and each individual has the option to extend their working years beyond the age of 68 years through a mutual agreement with their employer. As of 2021, the average retirement age in Sweden is 64.2 years across all professions (not limited to first-line managers in health care) [16].

In this study, we used a purposive sample of first-line managers who had resigned from their position in 2020 or later. We used purposive sampling to specifically target individuals with experience of first-line management who had recently resigned, as their perspectives were particularly valuable for our research. To recruit participants, we were given contact information by the region's human resources department when a first-line manager had given notice to leave their managerial position. Participants were recruited consecutively, but we faced some disruption in the inclusion due to the COVID-19 pandemic. Recruitment began through an e-mail containing written information about the study, sent before a potential participant left their position. Two weeks after sending the e-mail, the last author, an experienced qualitative researcher and registered nurse, contacted the potential participant to inquire if they had any questions, needed clarification, and would be willing to participate in the study. The last author had no previous relationship with any of the participants. In total, 25 first-line managers were invited to participate in the study. Out of these, 16 first-line managers agreed to participate, whereas the remaining nine either did not respond to the invitation or declined participation. The first-line managers who participated in the study represented a diverse selection from both primary care and inpatient care contexts. The sample consisted of 13 females and 3 males aged between 42 and 64 years (mean 55.5 years). Their experience as a manager ranged between 3.5 and 35 years (median 9 years, mean 10.8 years). Their most recent tenure as a first-line manager ranged from 1.5 to 30 years (median 4.25 years, mean 7.5 years).

### 2.3. Data Collection

Before conducting the interviews, the written information previously sent to the participant was repeated and written consent for participation in the study was obtained. Data were collected through individual semistructured interviews conducted by the last author between January 2020 and November 2022, with parts of the data collection taking place during the COVID-19 pandemic which occasionally affected the scheduling of the interviews. A semistructured interview guide was used to describe first-line managers' thoughts on and motives for leaving their position. Questions such as “What was the primary reason for leaving your role as a first-line manager?” and “How did you come to that decision?” were used. Follow-up questions, such as “Can you please tell me more?”, were used to gain a better understanding of their statements. The last author, a researcher with extensive experience of first-line management and leadership, conducted one-to-one interviews digitally or in person at a location chosen by the first-line manager being interviewed. Interviews lasted between 43 and 77 min and were recorded in digital audio files. These files were securely stored on a password-protected server. The first author, a registered nurse with some research experience, transcribed the interviews verbatim into Word files. According to Malterud et al. [17], the concept of information power can guide decisions on sample size in qualitative research. Information power refers to the idea that the more relevant information the sample holds in relation to the aim of the study, the fewer participants are needed. This is influenced by several factors, including the specificity of the sample, the narrowness of the study aim, the quality of the interview dialog, and the chosen analysis strategy. In our study, we had a narrow aim, focusing specifically on first-line managers who had left their position. We used purposive sampling to ensure that participants had direct experience of the phenomenon under study, and we were careful in selecting relevant participants and formulating interview questions that closely aligned with the aim of the study. We judged that a small number of interviews would be sufficient to achieve information power based on the aim, the specificity of the questions, and the quality of the dialog in the interviews, as described by Malterud et al. [17]. However, we took the opportunity to collect more data and conducted 16 interviews in total. We used purposive sampling to ensure specificity and relevance.

### 2.4. Data Analysis

The analysis started after all the interviews had been conducted and transcribed in Word files. We used specially designed tables in Word documents to sort the data in each step of the analysis process (see Supporting information 1 for examples). We used qualitative content analysis inspired by Graneheim et al. [18] to analyze the data. First, each transcript was read and listened to several times to achieve data immersion and to obtain a holistic understanding of the content. Based on the study aim, meaning units were identified and extracted into the analysis table. Second, these meaning units were condensed, meaning that the content was shortened while preserving the essential meaning. This step remained close to the participants' expressions and reflected the manifest content of the text. Each condensed meaning unit was then assigned a code that captured its core message in a concise form. Third, the codes were compared across the interviews and sorted in a new table based on similarities and differences. This comparative process enabled the clustering of codes into subthemes, which represent recurring patterns within the data. Through an interpretative process, these subthemes were further abstracted into one main theme, which represents a higher level of latent content, capturing the underlying meaning that runs through the subthemes. The entire process from meaning units to condensed units, to codes, subthemes, and ultimately theme entailed a gradual movement from the descriptive to the interpretative level, with increasing abstraction. The theme can be understood as the unifying “red thread” that gives deeper meaning to the participants' shared experiences, while the subthemes illustrate the diversity and nuance within these experiences [18]. Throughout the analysis process, the first author maintained an ongoing dialog with the last author, and all authors discussed the results until a consensus was reached, to improve the trustworthiness of the analysis.

### 2.5. Ethical Considerations

This study has been performed in accordance with the Declaration of Helsinki [19] and received ethical approval from the Swedish Ethical Review Authority, registration number 2019-04583. The participants were informed that participation was voluntary and that they could withdraw from the study at any time without providing any explanation. Participant and data confidentiality was guaranteed.

## 3. Findings

The theme “A tipping point for the unit and manager” was based on three subthemes that were interpreted from the first-line managers' experiences; see Table 1 for an overview.

The subtheme “Overwhelming challenges” includes first-line managers' descriptions of the difficulties they faced at work, which caused them to eventually reach a tipping point. The lack of influence, feeling unheard, lack of support, and resistance to them taking vacations created challenges in carrying out their assignment and potentially impacted their health.

The subtheme “Time to leave” related to reaching a tipping point in the first-line manager role as well as within the unit. First-line managers described reaching a point where it was time for them to move on. This could be due to personal reasons such as wanting to spend more time with their family, or the realization that they lacked the motivation to continue developing the unit and felt it was time for someone else to take over.

The subtheme “Bridging the gap” includes descriptions of first-line managers' actions once they had decided to resign. Here, the first-line managers focused on what could benefit their successor, the staff, and the unit as a whole.

Across all three subthemes, first-line managers mentioned having reached a tipping point for themself and/or for the unit. The “overwhelming challenges” led to a tipping point causing first-line managers to resign due to a unmanageable and unsustainable work situation, which had consequences for both them and their unit. When first-line managers who had managed a unit for a longer time resigned because it was “time to leave,” that had consequences for the unit. The consequences of their resignation were seen as a tipping point.

### 3.1. Overwhelming Challenges

Seven out of the 16 first-line managers in the study decided to leave their managerial role due to overwhelming challenges. The first-line managers stated that some challenges that arose in their day-to-day work could affect them negatively. Not having the authority to influence decisions or give suggestions to improve their work situation was described as an obstacle for first-line managers to fulfil their duties. This could lead them to leave their role out of frustration. Some said that their complaints about working conditions were ignored, despite having had several discussions with their second-line manager, and they felt powerless in the decision-making process.

I've had several conversations with my manager where I said, I don't even know if I want to continue in this role. And the reason is that—even though I'm the manager of my unit, I honestly don't feel like I have any real authority. Excuse my language, but it feels like I'm not allowed to decide a damn thing (IP15)

The lack of support and cooperation from the second-line manager was described as causing problems. As first-line managers, they had to implement the decisions made in the organization, even if they went against their own personal preferences. Feeling unheard and unable to influence their work situation could also force them to take sick leave if the workload became too heavy. It was stated that when there were changes in second-line management, including changes in managerial positions, there was often a decrease in the level of support experienced by first-line managers. The managers expressed a preference for full-time employment over time-based employment. Some first-line managers described not getting permission to take vacation days as they desired during the holiday period because there was a shortage of staff on the floor. This meant that they had to participate in care of patients—adding to their workload and stress due to performing two jobs.

Yeah, in the end it was just too much for me … Because I was, I was all alone. I didn't have anyone and … I didn't get a lot of support from the new second-line manager who came in later, so … (IP13)

The lack of influence and support and the resulting stress were made worse by the difficulty of dealing with staff who directed unkind words and behaviors toward the first-line manager. Managing such employees posed a major challenge, as it had a negative impact on their well-being. For example, some employees could insist that a first-line manager should not be allowed an extension in the position because they were dissatisfied with the manager's work. Some employees talked behind the manager's back or questioned their ability to give feedback, which affected self-confidence and behavior. Some first-line managers supported these individuals, saying that they were good people who were unhappy with their working conditions and were expressing dissatisfaction with the first-line manager simply as a representative of the employer. In situations where first-line manager colleagues also exhibited bad behavior and did not provide support, the work situation became even less sustainable. First-line managers who did not feel supported by their second-line manager perceived such behavior as being permitted among their colleagues. When adapting to working conditions that affected their well-being, some first-line managers experienced a sense of discomfort, as they felt they were not being true to themself. This was described as a feeling of dissatisfaction with how they performed their managerial duties, which increased stress levels. If stress persisted over a long period of time, it could lead to sleeping difficulties and anxiety. When first-line managers looked back, some said that their task seemed impossible. Despite their ambition to remain in their position, there was a limit to how much they could deal with.

Now, the thing is that nine out of ten come here and do a good job and behave well, but then we have a small group …//… where some people have an attitude and they talk me down a lot behind my back: what I was like as a person, what a bad job I did, it was mainly personal characteristics that were attributed to me. That, ehm … yeah, different… it was bullying the manager, really (IP3)

### 3.2. Time to Leave

Nine of 16 first-line managers left their managerial role because they felt it was time to leave. They had a plan for their exit and did not leave due to an overwhelming workload. They decided to resign for reasons such as lack of motivation, workload reduction, or prioritizing personal life. Some first-line managers felt it was time to do something else because they no longer had the same drive to develop the unit and believed someone new should take over. This change was perceived as beneficial for the staff, as they would get to hear a new perspective. Some first-line managers stated that they had planned to resign their managerial position as they wanted to prioritize their personal life and reduce their workload.

The reason I'm stepping down is probably that I'm… well, I'm turning 64. I guess I want to work a bit less, and it's also about wanting to spend more time with my grandchildren. You know, there's life outside of work too—and to me, that's important, of course (IP14)

Some first-line managers left their position because they possessed a skill or competence needed elsewhere or wanted to work on a new project. Those who had been first-line managers for many years typically looked back on that time with pride and joy.

If you aren't growing and aren't moving forward and … we didn't know what this was, but I felt that … no, the feeling just came over me that I was done with this, I was finished with this (IP8)

### 3.3. Bridging the Gap

Regardless of the reason that a first-line manager left, they aimed to deal with the tipping point for the unit by considering how the gap could be bridged for the unit and their successor. In cases where a first-line manager left their position because they felt it was time to leave, they had the opportunity to discuss with the second-line manager what qualities the new first-line manager should possess and how personnel responsibilities should be distributed within the unit. If there was a dialog between the first-line manager and the workplace, this could make things easier for the successor, through use of a transition phase and a plan for succession.

My manager and I had a dialogue about the successor, since she asked me what I thought about …//… what qualities are needed in the role (IP2)

There would usually be a period of around a month where the leaving first-line manager would remain available as backup and support. This type of phase-out and handover period was used to ensure a smooth transition. Some first-line managers chose to move to the background, to allow the new first-line manager to shape the unit without interference. First-line managers described this as beneficial for staff too, who would then actively seek support from the new manager. That was particularly helpful if the previous manager had been in the role for an extended period of time. In some cases, a washout period was said to ease the transition, especially for first-line managers who described themselves as wanting to be involved and influence all unit decisions and who struggled to step back. In these cases, the leaving first-line manager could also be available as support, but was less actively involved in the transition.

I'm not worried, not at all. It'll be fine and otherwise I'll just have to be somewhere and support them until they've figured it out, because it does take time to get into things as a new manager, I know that from experience, it takes, like, before you've landed in the job and understand, understand the operations (IP9)

Some first-line managers chose to leave their position due to overwhelming challenges. However, if they did not collaborate with their second-line manager to plan for their successor, they had limited ability to influence who their replacement would be. This was because they had not discussed the matter with their second-line manager. In some situations, the first-line manager described having concerns about how the successor could cope with the tasks as they lacked management experience. Second-line managers sometimes told first-line managers that they would have difficulty finding a suitable replacement. Some first-line managers did a lot to facilitate for their successor, even if they had quit because of the challenges of the managerial role. The first-line managers wanted to hand over a functioning unit and ensure that their successor had the greatest possible chance of success. They often described the process as “paving the way” for their successor.

My successor knows the job better now… she enters under different conditions—let's just say I've paved the way for her. So yes, I've paved the way (IP3)

## 4. Discussion

This study aimed to describe first-line managers' thoughts on and motives for leaving their position. Our result identified an overarching theme, “A tipping point for the unit and manager,” which encompassed three subthemes: overwhelming challenges, time to leave, and bridging the gap. Regardless of why first-line managers left their role, their doing so had a significant impact on their unit. In addressing these consequences, the first-line managers strove to ensure a smooth transition for the unit and their successor. The first type of reason for leaving the position was related to overwhelming challenges in the managerial role, such as lack of support and challenges with a limited mandate that affected working conditions. Dealing with unkind words and behaviors within the staff team became more complicated when the immediate superior did not provide support. Such factors caused stress and frustration, which ultimately led some first-line managers to resign from their position because they felt their work situation was unmanageable and unsustainable. The second type of reason involved feeling that it was time to leave the managerial role. Some managers wanted a better work–life balance, whereas others felt that they had reached a dead end in developing the unit. The resignation of a first-line manager who had left a mark on a unit and worked there for an extended period had consequences for the unit, creating a tipping point. Regardless of why they chose to leave their position, the first-line managers often worked to facilitate for their successor. By bridging the gap, they thought they could make it easier for the unit and the successor to deal with their resignation. To ensure a smooth transition for their successor and the unit as a whole, they aimed to make it easier for all those affected to adapt to the change. Their focus was on addressing the aftermath of their resignation in a way that benefited their successor, the staff, and the unit.

### 4.1. Overwhelming Challenges

Due to their responsibilities, first-line managers faced overwhelming challenges in their work. Studies have shown that they often feel excluded from decision-making processes [11, 20, 21]. This can lead to low job satisfaction and result in a decision to leave the position [5, 6]. In our findings, first-line managers stated that they often lacked authority to influence decisions and give suggestions on how their work situation could be improved. Complaints about working conditions were ignored, leading to dissatisfaction and a feeling of not being heard. These findings are comparable to those of previous studies [11, 21], indicating a need for more active participation of first-line managers in decision-making processes. To mitigate these challenges, it is important to empower first-line managers with greater authority and provide them with the support necessary to ensure successful management of their units. This could enable them to be more effective managers, as shown by previous research [11, 21]. It is recommended to involve first-line managers more actively in decision-making processes [11], as this might result in the choice to remain in the managerial role [5, 6].

First-line managers often face challenging working conditions characterized by conflict, intrigues, and a lack of trust, making it difficult for them to maintain integrity and effectiveness. In regard to retainment, Arakelian and Rudolfsson [22] have highlighted the important role that trust in superiors has for first-line managers. Fostering a culture of transparency and open communication within an organization can help build trust between first-line managers and their superiors, ultimately enhancing performance and creating more positive working conditions. It is important to ensure effective cooperation and coordination between all parties involved in an organization while maintaining reasonable workloads for managers.

According to Tuna and Kahraman [23], harassment can manifest in various ways and can have severe impacts on the psychological and physical well-being of first-line managers. Based on our findings, it appears that even a small number of employees with a negative attitude can have negative effects on the well-being of first-line managers. Toxic behavior includes talking behind a manager's backs and questioning their abilities, which can result in both issues at work, such as poor performance and feelings of incompetence, and personal problems like exhaustion, unhappiness, and sleeping difficulties. The challenges faced by the interviewed first-line managers were consistent with those described by Tuna and Kahraman [23].

### 4.2. Time to Leave

Some first-line managers resigned as they felt it was time to leave because they had achieved all they could in their role. This affected their motivation to continue developing the unit and could lead them to feel that it was time for someone else to take over. They realized that their initial drive and motivation had decreased and thought a fresh perspective would benefit the unit's continued development. This decision-making process indicates a broader understanding of the idea that a first-line manager can benefit from having new ideas and enthusiasm, which a new manager can bring. Additionally, many first-line managers prioritized personal life aspects, such as reducing their workload or achieving a better work–life balance. Some left their position to utilize their skills in another context or to start new projects, indicating a desire for career advancement or change. These findings relate to the research conducted by [3, 4], highlighting that common reasons for first-line managers' resigning include career advancement and retirement. The alignment with these studies underlines the importance of understanding managerial turnover factors. It also highlights the need for organizations to address these factors, to maintain effective leadership and continuous unit development.

### 4.3. Bridging the Gap

By bridging the gap, first-line managers showed their respect for the staff at the unit, wanting to ensure that they were left in good hands. Having a plan for a successor also shows good leadership and foresight on the part of the first-line manager, allowing for a smooth transition and ensuring that the unit can continue to operate effectively without disruption. Our findings suggest that first-line managers often create succession plans for their successors, even if the organization does not give instructions on how to do this. This was more common among individuals who left their job because they felt it was time to leave and prioritized a more balanced approach to work and leisure time. However, it was also seen among those who quit because the demands of their managerial duties became too overwhelming. These findings highlight the fact that first-line managers have a strong desire to ensure the functionality of their unit. This is understandable, as they have likely invested time and effort into building and maintaining the unit. A study conducted by Pilat and Merriam [24] showed that new first-line managers usually do not get proper organizational onboarding, though they can get support and guidance from their colleagues in the management team. This underlines the importance of having a supportive work environment, as it helps new first-line managers feel prepared and confident in their role. Interestingly, previous research has highlighted potential barriers to succession planning, including a reluctance among current first-line managers to share their knowledge and experience with potential successors due to feelings of insecurity or egotism [25]. In contrast, our findings indicate that many first-line managers actively engaged in facilitating a smooth transition and willingly supported their successors, suggesting that such barriers may not always be present in practice. Previous studies have also emphasized the value of structured onboarding programs, including mentorship and dedicated time for preparation, to support new first-line managers and reduce turnover [25, 26]. While our study did not focus on organizational onboarding practices, the willingness among departing first-line managers to “pave the way” for their successors highlights the importance of informal transition efforts and could inform future development of more formalized support systems.

### 4.4. Theoretical Perspective

Our findings can also be understood from a theoretical perspective, using the framework of health-promoting and sustainable leadership developed by Dellve and Eriksson [27]. This framework includes five levels, of which three can be related to our results: individual, micro, and macro. The individual level focuses on employee well-being, motivation, job satisfaction, competence, experience, attitudes, capacity and behavior. The microlevel pertains to the immediate work context in which employees perform their tasks, as well as their current workplace. The macrolevel encompasses societal factors such as work environment legislation and pension systems, which organizations must take into account and comply with. The results of our study reveal that the reasons why managers left their managerial position could usually be attributed to individual or microlevel factors. First-line managers who left their role because they felt it was time to leave most likely had reasons at the individual level, as the focus was on their own well-being. First-line managers who faced overwhelming challenges and decided to leave their position because of this likely had reasons at the microlevel. This pertains to the work context and workplace, including the challenges faced by first-line managers in their day-to-day role. The macrolevel can be connected to the fact that first-line managers often wanted to make the transition easier for their successor, despite a lack of organizational support.

## 5. Strength and Limitations

The varying levels of openness exhibited by the participants in this study may have impacted the data collected during the interviews. Those who were more forthcoming and open in their responses may have provided greater insights into their motives for resigning, which may have led to a deeper understanding of their issues. However, those who were more guarded in their responses perhaps especially those who were close to retirement may have expressed their opinions more subtly, for example, by using vague or indirect language such as saying “it just felt like the right time” rather than explicitly stating specific challenges or dissatisfaction. It is possible that such phrasing reflects a personal process of reflection or emotional resolution, where participants had already come to terms with their work situation and framed their departure as a natural transition rather than a reaction to specific difficulties. This may have affected the overall balance of perspectives in the study and the nuances of the interpretations made. To promote the credibility, dependability, and transferability of qualitative research, certain factors must be taken into consideration [18]. The data collection and analysis were aligned with the research objective, to enhance credibility. The inclusion of participants who accurately represented the phenomenon under study also enhanced credibility. Dependability was promoted by ensuring constant dialogs among the researchers. Additionally, the subthemes and theme were discussed until consensus was reached. The data were collected by the same person throughout the collection process which enhanced consistency in how the interviews were conducted and how follow-up questions were posed. The interviewer had extensive experience in conducting qualitative interviews, which contributed to a structured yet flexible approach that encouraged participants to share their thoughts openly. However, the data were collected during a pandemic, which caused a delay in the collection process and might have affected dependability. Transferability was achieved by presenting representative quotes and providing information regarding the context, data collection, and data analysis.

## 6. Conclusions

The work situation in which the first-line managers handled a number of diverse and at times challenging tasks could be overwhelming, in particular when they lacked authority and support. Some interviewed managers chose to leave their position due to frustration and negative impact on well-being. Factors in social life, such as wanting more time for family and life outside work, were also cited as reasons for leaving their role. The desire for new challenges in their work life influenced some managers. Some lacked the motivation to develop their unit and therefore chose to leave their role. In the process of leaving the position as first-line manager, the participants considered and planned measures to implement, so as to ensure a smooth transition for their successor, the staff, and the unit.

Regardless of why a first-line manager leaves their position, their action becomes a tipping point for the unit, with widespread impact. It is of the utmost importance that hospital management supports and manages the resulting changes in a professional manner. Giving the first-line manager support to face their new situation and giving the successor, the unit staff and the organization good preconditions for the future is a managerial responsibility of strategic importance. Fostering a work environment which empowers first-line managers with sufficient authority in their role is crucial for hospital management in retainment of first-line managers.

## Figures and Tables

**Table 1:** Overview of the subthemes and theme describing first-line managers' thoughts on and motives for leaving their position.

Subtheme	Theme
Overwhelming challenges	A tipping point for the unit and manager
Time to leave	
Bridging the gap	

## Data Availability

Due to the nature of the research, legislation, and ethical approval restrictions, supporting data are not available. To preserve the privacy of the involved individuals, in accordance with the European General Data Protection Regulation, the raw data generated in this study are not available.

## Supporting Information

The supporting information illustrate how meaning units were condensed and coded, providing insight into the analytical procedure (see Supporting information 1, e.g.), and how the codes were subsequently grouped into subthemes that led to the overarching theme.
