# Supplementary material for: Thoughts on and Motives for Leaving the First-Line Manager Position: A Qualitative Study
Source: J Nurs Manag. 2025 Aug 16;2025:9906205. doi: 10.1155/jonm/9906205 (PMC12374806; doi:10.1155/jonm/9906205)
Supplement: Supporting Information — Additional supporting information can be found online in the Supporting Information section. [file 9906205.f1.docx]

Table 1. Examples of meaning units, condensed meaning units and codes.

| Meaning unit | Condensed meaning unit | Code |
| --- | --- | --- |
| Yes, it ended up being too much for me. … Because I was, I was very alone. I didn't have anyone and… I didn't have very good support from the new manager that came then that was it. | It ended up being too much for me. Because I was very alone. I didn't have anyone, and I didn't have very good support from the new manager. | In the end, it was too much for me, and I was alone and didn’t get much support from my new manager. |
| The last time I extended I signed for 2 years I said I'm signing for 2 years and then I don't want to continue but then I want to do something else | The last time I extended I signed for 2 years I said I'm signing for 2 years and then I don't want to continue, then I want to do something else | The last time I extended I signed for 2 years, then I wanted to do something else. |
| Yes, it should be [a well-functioning unit]. It feels good because, as I mentioned, we've had many people leaving recently, and it's important to have sufficient staff during transitions. This way, the new manager won't have to start from scratch with recruiting. I want to make sure everything is prepared so that the unit is in good condition, [laughs]. Now, it feels good to leave under these circumstances. There are also considerations for the new person stepping in. | Yes, it should be [a well-functioning unit]. It feels good, as I mentioned, we've had many people leaving recently, and it's important to have sufficient staff during transitions. This way, the new manager won't have to start from scratch with recruiting. I want to make sure everything is prepared so that the unit is in good condition. It feels good to leave under these circumstances. | It should be a well-functioning unit. I wanted to ensure it was in good condition beforehand, providing a smooth transition for the new manager. |

Table 2. Examples of codes, subtheme and theme.

| Theme | A tipping point for the unit and manager | | |
| --- | --- | --- | --- |
| Subthemes | Overwhelming challenges | Time to leave | Bridging the gap |
| Codes | In the end it was too much for me, I was alone and had no support from my new manager | The last time I extended I signed for 2 years, then I wanted to do something else. | It should be a well-functioning unit. I wanted to ensure it was in good condition, providing a smooth transition for the new manager. |
